# Short-chain fluorescent tryptophan tags for on-line detection of functional recombinant proteins

**DOI:** 10.1186/1472-6750-12-65

**Published:** 2012-09-21

**Authors:** Eva-Maria Siepert, Esther Gartz, Mehmet Kemal Tur, Heinrich Delbrück, Stefan Barth, Jochen Büchs

**Affiliations:** 1 Department of Experimental Medicine and Immunotherapy, Institute of Applied Medical Engineering, Helmholtz Institute of RWTH Aachen University & Hospital, Pauwelsstr 20, 52074 Aachen, Germany; 2 AVT. Biochemical Engineering, RWTH Aachen University, Worringerweg 1, 52074, Aachen, Germany; 3 Department of Pharmaceutical Product Development, Fraunhofer Institute for Molecular Biology and Applied Ecology (IME), Forckenbeckstr 6, 52074, Aachen, Germany; 4 Institute of Molecular Biotechnology, RWTH Aachen University; c/o Fraunhofer IME, Forckenbeckstr 6, 52074, Aachen, Germany; 5 Institute of Pathology, University Hospital Giessen and Marburg GmbH (UKGM), Langhansstr 10, 35392 Giessen, Germany

**Keywords:** Tryptophan tag, On-line monitoring, Microtiter plate, Fluorescence measurement, *Escherichia coli* protein expression, Small scale fermentation

## Abstract

**Background:**

Conventional fluorescent proteins, such as GFP, its derivatives and flavin mononucleotide based fluorescent proteins (FbFPs) are often used as fusion tags for detecting recombinant proteins during cultivation. These reporter tags are state-of-the-art; however, they have some drawbacks, which can make on-line monitoring challenging. It is discussed in the literature that the large molecular size of proteins of the GFP family may stress the host cell metabolism during production. In addition, fluorophore formation of GFP derivatives is oxygen-dependent resulting in a lag-time between expression and fluorescence detection and the maturation of the protein is suppressed under oxygen-limited conditions. On the contrary, FbFPs are also applicable in an oxygen-limited or even anaerobic environment but are still quite large (58% of the size of GFP).

**Results:**

As an alternative to common fluorescent tags we developed five novel tags based on clustered tryptophan residues, called W-tags. They are only 5-11% of the size of GFP. Based on the property of tryptophan to fluoresce in absence of oxygen it is reasonable to assume that the functionality of our W-tags is also given under anaerobic conditions. We fused these W-tags to a recombinant protein model, the anti-CD30 receptor single-chain fragment variable antibody (scFv) Ki-4(scFv) and the anti-MucI single-chain fragment variable M12(scFv). During cultivation in Microtiter plates, the overall tryptophan fluorescence intensity of all cultures was measured on-line for monitoring product formation via the different W-tags. After correlation of the scattered light signal representing biomass concentration and tryptophan fluorescence for the uninduced cultures, the fluorescence originating from the biomass was subtracted from the overall tryptophan signal. The resulting signal, thus, represents the product fluorescence of the tagged and untagged antibody fragments. The product fluorescence signal was increased. Antibodies with W-tags generated stronger signals than the untagged construct.

**Conclusions:**

Our low-molecular-weight W-tags can be used to monitor the production of antibody fragments on-line. The binding specificity of the recombinant fusion protein is not affected, even though the binding activity decreases slightly with increasing number of tryptophan residues in the W-tags. Thus, the newly designed W-tags offer a versatile and generally applicable alternative to current fluorescent fusion tags.

## Background

Complex gene and protein libraries allow the identification of pharmaceutically relevant drug targets by initial screening [1] in Microtiter plates (MTPs) followed by scaling up to industrial production. MTPs are used for the high throughput parallel characterization of microbial cultures under identical conditions. Furthermore, instruments based on the recently developed BioLector® technology [2] allow fermentation parameters such as cell growth, dissolved oxygen tension (DOT) and pH to be monitored on-line, using specially adapted MTPs [3].

The production of recombinant proteins during cultivation is often monitored using fluorescent fusion proteins [4]. Common fusion partners involve green fluorescent protein (GFP) [5] and its derivatives such as yellow fluorescent protein (YFP), as well as fluorescent proteins containing flavin mononucleotides (FMNs) (also called FMN-based fluorescent proteins (FbFPs)) such as the evoglow® blue light receptor [6,7]. The fluorescent proteins are detected using non-invasive, specific and sensitive devices that monitor product formation and localization *in vivo*. They represent the state of the art for screening and bioprocess optimization.

One drawback of fluorescent proteins based on GFP is their relatively high molecular mass (26.9 kDa) compared to typical target proteins. It is discussed in literature that this might impose stress on host metabolism during fermentation. GFP and its derivatives also require an aerobic environment with sufficient cellular oxygen to form the fluorophore [5,7-9]. Furthermore, detection of this fluorophore is completely inhibited in an oxygen-limited or anaerobic environment. The on-line measurement of GFP is feasible, but is not ideal in fermentation systems that yield active protein products, particularly those with a low molecular weight. The FMN-based blue light receptor evoglow® was developed as an alternative to GFP because it is smaller (42% by molecular weight) and is not oxygen-dependent [6,7,10]. Although this is an improvement, even smaller fluorescent protein tags may be preferable because they would have a minimal impact on host strain metabolism allowing more resources to be committed to the production of the recombinant target protein.

One potential solution is the development of tags based on the auto-fluorescent properties of aromatic amino acids such as tryptophan (W), tyrosine (Y) or phenylalanine (F) [11]. Here, delocalized π-electrons in the aromatic ring structures are excited to higher energy states when exposed to certain wavelengths of light, and emit fluorescence in the UV range when they return to their ground state. All three amino acids depict hydrophobic properties. Tryptophan is the best choice for fluorescent tags because it has a relatively high quantum yield and a larger Stokes shift than the other aromatic amino acids (~70 nm), with an excitation maximum at 280 nm and an emission maximum at 350 nm. Its fluorescence is highly sensitive to the properties of the surrounding environment (i.e. polarity) and neighboring amino acids [12]. Therefore, the spectral characteristics of tryptophan can be enhanced in the presence of tyrosine [13]. Most proteins are statistically likely to contain tryptophan but the number and distribution of residues are variable [14]. Therefore, it should be possible to distinguish between untagged proteins and those carrying a specific tryptophan-based fusion tag (here described as a W-tag). Tags with tryptophan residues have previously been used to improve protein isolation by creating a hydrophobic affinity patch rather than a fluorescent label [15,16]*.*

We designed a series of W-tags containing different numbers of tryptophan residues within a hairpin/β-sheet motif to determine whether they would act as unobtrusive fusion markers for the quantitative real-time measurement of product formation. The W-tag size corresponds to only 5-11% of the size of GFP and 12-27% of the blue light receptor, respectively. The tags were introduced into the pET expression vector [17] as in frame fusions with two single-chain antibody fragments: (1) as proof of concept, the anti-CD30 antibody Ki-4(scFv) [18], allowing functional analysis in the CD30-positive L540cy human Hodgkin’s lymphoma cell line [18]; and (2) for additional measurements the anti-MucI antibody M12(scFv) [19] present on the human mamma carcinoma cell line MCF7.

Ki-4(scFv) has a tryptophan content of 2.88% (six residues), challenging our detection system to distinguish between tagged and non-tagged proteins on the basis of fluorescence intensity (tryptophan content of different W-tagged Ki-4(scFv) fusion proteins: W1-Ki-4(scFv) = 2.84%, W2-Ki-4(scFv) = 3.06%, W3-Ki-4(scFv) = 3.42%, W4-Ki-4(scFv) = 3.80%, W5-Ki-4(scFv) = 4.18%). Therefore, we set out to determine the suitability of W-tags for non-invasive on-line monitoring using an adapted BioLector filter device to detect biomass via scattered light and product formation via tryptophan fluorescence intensity. We also tested the binding activity of the Ki-4(scFv) antibody carrying different types of W-tag by flow cytometry. We discuss the performance of our novel tags in comparison to conventional (GFP) and FMN-based fluorescent proteins. The newly designed W-tags present an alternative and very promising tagging method to GFP, its derivatives and the evoglow® blue light receptor.

## Results

### W-tag design and vector assembly

**Figure 1:**
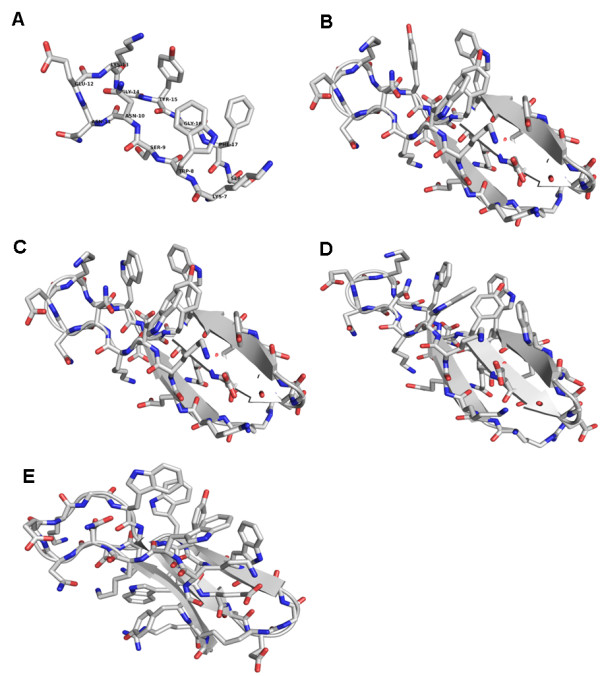
**Illustration of 3D amino acid sequence structures of all five W-tags.** (**A**) W1-tag, (**B**) W2-tag, (**C**) W3-tag, (**D**) W4-tag and (**E**) W5-tag; all structures display the amino acid residues on molecular basis showing the presumed/calculated in- and outward orientation of the amino acids in the protein loop used for the W-tag design.

We modeled short, optically active W-tags by integrating the auto-fluorescent amino acid tryptophan (W) into a naturally occurring β-sheet motif from the *Bacillus caldolyticus* cold shock protein (*Bc-Csp*) [20] obtained by screening the Protein Data Bank (PDB) for small motifs with the appropriate distribution of hydrophobic amino acids. *Bc-Csp* [PDB: 1C9O] is a small protein (66 amino acids) comprising two β-sheets that form a β-barrel. The first β-sheet (30 amino acids) consists of three β-strands connected by two loops, with a hydrophobic core and one tryptophan and two phenylalanine residues exposed on the surface. Starting from the third residue, the sequence was systematically mutated *in silico* to increase the number of tryptophan residues. We used CHARMM ( http://www.CHARMM.org) in Discovery Studio ( http://www.accelrys.com) to minimize the free energy of each model, and selected the models with the lowest free energy for each number of substituted tryptophan residues to construct the corresponding W-tags (Figure 1).

**Figure 2:**
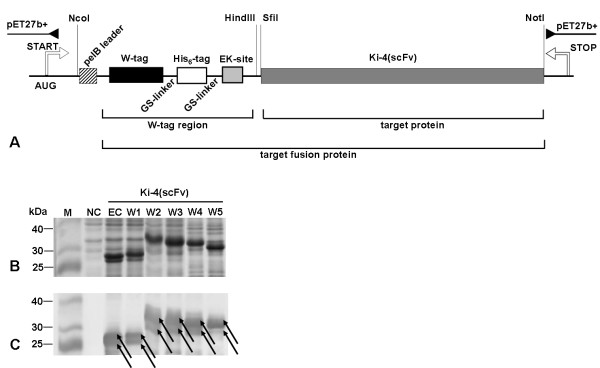
**Cloning scheme for Wx-Ki-4(scFv) fusion protein constructs, SDS-PAGE and Western blot analysis of Wx-Ki-4(scFv) expression.** (**A**) Plasmid map of pET-Wx-Ki-4(scFv). The plasmid backbone contains a kanamycin resistance gene (kan), a pBR322 origin of replication and the lactose repressor gene (*lacI*). The Ki-4(scFv) sequence is genetically linked to the W-tag. The schematic structure of the Wx-Ki-4(scFv) insert in the expression cassette consists of the *pel*B signal peptide inducible with IPTG via the *lac* operator. The W-tag is fused to a His_6_-tag by a GS-linker and the Ki-4(scFv) is linked to the His_6_-tag through a cleavable enterokinase site. Restriction sites are also shown. (**B**) SDS-PAGE: lane 1 – Prestained Broad Range Protein Marker (NEB, USA), lane 2 – negative control (NC) (pET-27b+ vector), lane 3 – expression control (EC) (Ki-4(scFv)), lane 4 – W1-Ki-4(scFv), lane 5 – W2-Ki-4(scFv), lane 6 – W3-Ki-4(scFv), lane 7 – W4-Ki-4(scFv), lane 8 – W5-Ki-4(scFv). (**C**) Western blot of proteins detected with an anti-poly-His antibody, a goat anti-mouse IgG peroxidise conjugated antibody and DAB. In figures (**B**) and (**C**) the proteins are detected as double bands. The lower band (25-30 kDa) corresponds to the calculated weight of the protein, the higher band (30-35 kDa) includes the uncleaved *pel*B leader peptide.

Five different W-tags were developed, containing between one and five tryptophan residues (accession numbers are: W1-tag [GenBank: JN107996], W2-tag [GenBank: JN120907], W3-tag [GenBank: JN120908], W4-tag [GenBank: JN120909] and W5-tag [GenBank: JN120910]. These were fused in frame to the Ki-4(scFv) sequence using the pET-27b+ derived [21] pET-Ki-4(scFv) expression vector already carrying a gene encoding the CD30-specific single-chain antibody fragment Ki-4(scFv), a *pel*B leader for export to the periplasm and a His_6_-tag for immunodetection and purification (Figure 2A). The resulting vector series was named pET-Wx-Ki-4(scFv), where x refers to the number of tryptophan residues in the W-tag. The W-tags were approximately three times larger than the His_6_-tag. Table 1 gives an overview of the molecular sizes of the different W-tags and of the W-tagged fusion proteins. We used pET-Ki-4(scFv) without W-tags as an expression control (EC) for background auto-fluorescence, because the Ki-4(scFv) sequence already contains six tryptophan residues. We also used the empty pET-27b+ as a true negative control (NC). A homologous series of plasmids was created substituting the Ki-4(scFv) sequence with that of the M12(scFv).

**Table 1:** Five different variations of the W-tag, stating the number of tryptophan residues per construct, construct name, total number of aromatic amino acids (tryptophan, tyrosine and phenylalanine) as well as the size of the tag region and fusion protein in kDa

**Name**	**Aromatic amino acids**	**Fusion protein in kDa**	**W-tag in kDa**
EC	0	24.16	1.73
W1	1W 1Y 1F	25.79	3.36
W2	2W 3Y 0F	27.44	5.01
W3	3W 2Y 0F	27.46	5.03
W4	4W 1Y 0F	27.49	5.06
W5	5W 3Y 0F	28	5.57

### Protein expression

The five Wx-Ki-4(scFv) constructs plus controls were expressed in *E.coli* BL21 Rosetta 2 (DE3), and expression was verified by testing the bacterial crude lysate by sodium dodecylsulfate polyacrylamide gel electrophoresis (SDS-PAGE) followed by immunoblot analysis against the His_6_-tag (Figure 2B and C). We detected intense double bands ranging from 25 to 35 kDa, confirming the over-expression of the different antibody fusion proteins with and without the *pel*B leader peptide. The double bands occurred due to the incomplete cleavage of the *pel*B leader when the fusion protein is transported from the cytoplasm to the periplasmic space. The identity of each protein band was confirmed by mass spectrometry (data not shown).

The fusion proteins tagged with the W-tags W2 to W5 were found only in the cell pellet fraction not in the supernatant, whereas the untagged antibody (EC) and the W1 fusion protein were partially secreted and found in both the supernatant and cell pellet (data not shown).

### Fermentation and on-line fluorescence intensity measurements in MTPs

**Figure 3:**
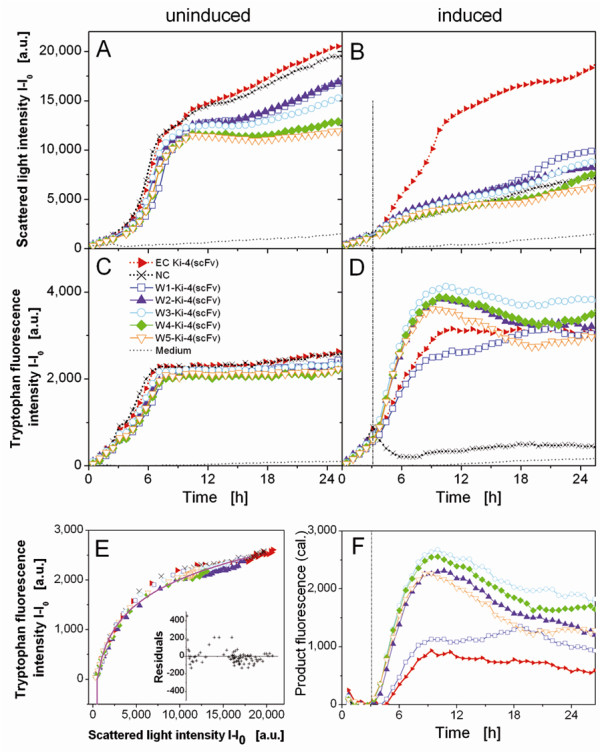
**On-line detection of biomass formation and production of W-tag labelled Ki-4 (scFv).** The intensities of (**A**,**B**) the scattered light (620 nm) and (**C**,**D**) tryptophan fluorescence (280/350 nm excitation/emission) were measured for the non-induced and induced cultures (induction with 1 mM IPTG is shown by the vertical broken line after 3.2 h). (**E**) For the non-induced cultures (no product), tryptophan fluorescence is plotted versus scattered light intensity (symbols). The appropriate fit shown as a continuous line (power function: fluorescence intensity_calc_ = 4780·[scattered light intensity_meas_]^0.077^-7715). The plot of the residuals between the calculated and the measured fluorescence is displayed within the diagram as an inset box. (**F**) Product fluorescence intensity calculated by subtracting the biomass fluorescence of the non-induced cultures from the total fluorescence of the induced cultures.

We used an adapted BioLector® device to measure biomass and tryptophan fluorescence simultaneously in induced and non-induced cultures over a period of 25 h (Figure 3A-D). Biomass was determined by measuring the scattered light signal at 620 nm [3], whereas tryptophan was excited at 280 nm and its emission was measured at 350 nm. The mean values for four parallel induced cultures and four parallel no induced cultures representing each Wx-Ki-4(scFv) fusion protein as well as the EC and NC constructs are shown in Figure 3A-E. The relative percentage deviation was less than 6% (data not shown).

The scattered light signals in the non-induced cultures (Figure 3A) increased during exponential growth phase and were comparable for all constructs up to 7.5 h, when the glucose was almost depleted (data not shown). These signals then declined until 10 h, when RAMOS (Respiration Activity Monitoring System) analysis confirmed that overflow metabolite acetate was fully metabolized (data not shown). The scattered light signals increased from 10 h onwards in the EC and NC cultures and, after a short stable period, also in the other cultures (Figure 3A) probably reflecting morphological changes of the bacteria [22]. The tryptophan fluorescence curves of the non-induced cultures were similar to the corresponding scattered light curves (Figure 3C). Remarkably, the tryptophan fluorescence curves reached a plateau after 7.5 h, confirming there is no further growth when the bacteria switch from consuming glucose to the overflow metabolite acetate. The similarity between the scattered light and tryptophan fluorescence curves shows that both signals provide data about biomass formation, which we discuss later in more detail.

For the non-inoculated wells, containing pure medium as a control, a slight increase for scattered light and tryptophan fluorescence signals was observed (Figure 3A-D). No cell growth was detected towards the end of the experiment (no pellet after centrifugation).

The induction of gene expression with 1 mM IPTG in the early exponential phase (after 3.2 h of cultivation) had a profound impact on the resulting growth curves. There was only a moderate increase in biomass formation (Figure 3B) but the tryptophan fluorescence curves showed a rapid increase in fluorescence in all but the NC cultures (Figure 3D). After 9-10 h, when the glucose was depleted, the tryptophan fluorescence intensity declined in the induced cultures.

In the non-induced cells, the correlation between increase of biomass (scattered light signal intensity) and tryptophan fluorescence intensity reflects the presence of tryptophan in the biomass (Figure 3E). The tryptophan fluorescence is plotted as a function of the scattered light where all data points coincide more or less with one another. This means that all five W-tag variants behave in a similar manner in terms of fluorescence vs. scattered light intensity in no induced cultures. The correlation between tryptophan fluorescence and scattered light intensity can be described by a power function (fluorescence intensity_calc_ = 4780·[scattered light intensity_meas_]^0.077^-7715). Consequently, the tryptophan fluorescence in the non-induced cultures is not dependent on the W-tag variant, but only on the scattered light intensity and, hence, the amount of cells.

In the induced wells, the overall tryptophan fluorescence signal is the sum of the fluorescence signal from the biomass and the fluorescence signal from the W-tagged fusion protein. The partial fluorescence signal from the tagged fusion protein can, therefore, be determined by subtracting the tryptophan fluorescence from the biomass (calculated using the power function as shown above) from the overall tryptophan fluorescence. If plotted against time (as shown in Figure 3F) it is clear that the fusion protein begins to accumulate only after induction. The fluorescence intensity then increases until 10 h of cultivation and declines thereafter, perhaps reflecting protein degradation. The EC construct also generates tryptophan fluorescence because it contains six tryptophan residues, but the signal intensity is substantially lower than that of the W-tagged proteins. We observed that an increasing number of tryptophan residues in the W-tags does not perfectly result in a proportional increase in fluorescence intensity. The reason for this is not yet clear. It may be attributed to different amounts of total fusion protein produced, giving a signal not consistent with the numbers of tryptophan in the W-tags. This issue has to be quantified by future investigations.

### Two-dimensional fluorescence intensity scans of crude cell extracts

**Figure 4:**
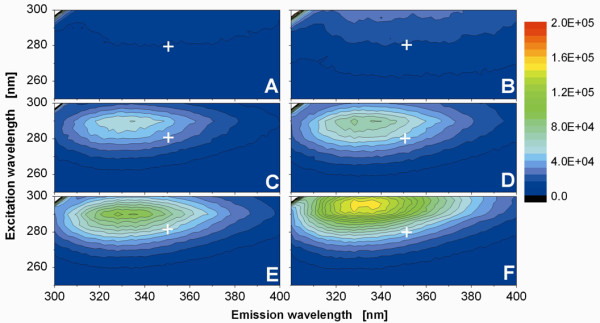
**Two-dimensional fluorescence intensity scan of concentrated Ki-4(scFv) tagged with different Wx-tags.** Depicted are fluorescence measurements of (**A**) an empty well, (**B**) 100% PBS buffer, (**C**) EC Ki-4(scFv), (**D**) W1-Ki-4(scFv), (**E**) W2-Ki-4(scFv) and (**F**) W3-Ki-4(scFv). For each well, the concentration of the Ki-4(scFv) and Wx-Ki-4(scFv) fusion proteins amounts to 3 μg/mL, harvested after 10 h cultivation. The 2D-scan shows that the increased fluorescence depends on the number of tryptophan residues in the tag. The empty well and PBS buffer do not generate significant signals. The cross in the diagram denotes the wavelength combination (280/350 nm excitation/emission) applied for the measurements.

To ensure that the W-tagged proteins maintained their ability to fluoresce after extraction and solubilization, crude extracts from cells expressing the W1-Ki-4(scFv), W2-Ki-4(scFv), W3-Ki-4(scFv) and EC constructs were analyzed by 2D fluorescence intensity scanning in a suitable MTP. Tryptophan fluorescence was excited between 250-300 nm and the resulting emission was detected in a range from 300-400 nm, respectively in 2 nm steps. In the MTP wells containing crude extracts the fluorescence intensity correlated with the number of tryptophan residues in the tag, whereas no fluorescence was detected in empty wells or wells containing pure PBS (Figure 4). Whereas the EC, W1-Ki-4(scFv) and W2-Ki-4(scFv) crude extracts generated maximum fluorescence values at comparable excitation/emission wavelengths (290/338 nm), there was a shift to 296/330 nm for the W3-Ki-4(scFv) crude extract.

### Flow cytometry analysis of antibody binding

**Figure 5:**
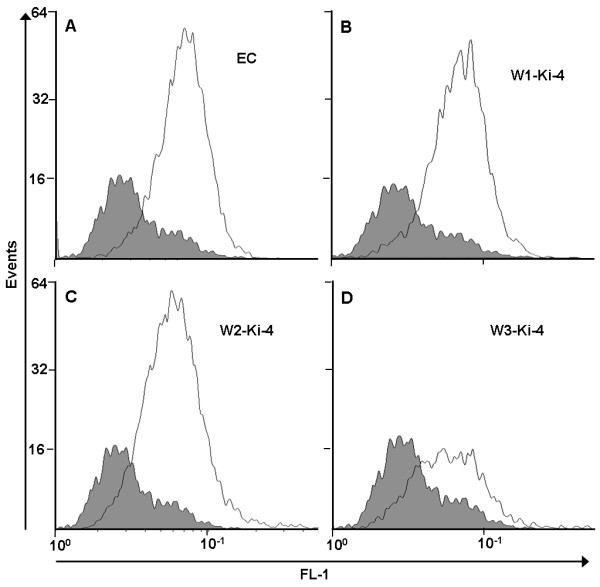
**Flow cytometric binding analysis of Wx-Ki-4(scFv) fusion proteins on L540cy cells.** (**A**) Ki-4(scFv), (**B**) W1-Ki-4(scFv), (**C**) W2-Ki-4(scFv) and (**D**) W3-Ki-4(scFv). The black curve represents L540cy cells incubated with only the secondary antibody (background). The grey curve represents the different versions of the Wx-Ki-4(scFv) proteins. The signal shift to the right means increased fluorescence intensity in relation to the black curve as the antibody binds to the CD30 receptor.

The binding activity of the Wx-Ki-4(scFv) fusion proteins was tested by flow cytometry. We found that the W1-Ki-4(scFv), W2-Ki-4(scFv) and the W3-Ki-4(scFv) fusion proteins as well as the untagged EC antibody were able to bind CD30-positive L540cy cells (Figure 5A-D) although the fusion protein variants showed a slightly lower statistical value for the mean fluorescence intensity (MFI) than the EC (Table 2). This resulted in a slightly lower Alexa Fluor 488 signal and indicated that binding between the fusion proteins and CD30 is slightly weaker than the corresponding interaction involving untagged Ki-4(scFv). The slight decrease of fluorescence signal, illustrated by the MFI and geometrical mean (%G-mean), may result from the binding of the secondary antibody to the His_6_-tag of the fusion proteins. It is possible that the His_6_-tag is partially obscured by the W-tag or that the W-tag folding itself causes this change (Table 2).

**Table 2:** Mean fluorescence intensity (MFI) values, marginally reduced when compared to MFI of 100% of Ki-4(scFv) and geometrical mean of the fusion proteins, displaying the same tendency

**(scFv) protein**	**Mean fluorescence intensity**	**% G-mean**
Ki-4(scFv) (EC)	7.13	100
W1-Ki-4(scFv)	6.67	93.6
W2-Ki-4(scFv)	6.73	94.4
W3-Ki-4(scFv)	6.69	93.8

### Competitive flow cytometry analysis

**Figure 6:**
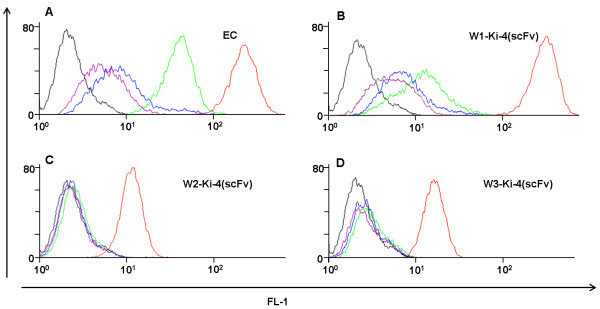
**Competitive flow cytometry of Ki-4(scFv), W1-Ki-4(scFv), W2-Ki-4(scFv), W3-Ki-4(scFv) against a monoclonal Ki-4 full flength antibody.** (**A**) Ki-4(scFv), (**B**) W1-Ki-4(scFv), (**C**) W2-Ki-4(scFv) and (**D**) W3-Ki-4(scFv). The black curve displays the measurement of the L540cy cells incubated with the secondary antibody α-His AlexaFluor 488 (Qiagen, Germany), the red curve presents the binding measurement of the Wx-Ki-4(scFv) protein detected via the α-His AlexaFluor 488 antibody without competitive addition of the monoclonal Ki-4 full length antibody. The green curve displays the fluorescence intensity of the Wx-Ki-4(scFv) proteins after adding and co-incubation with 0.5 μg Ki-4 full length antibody. The blue curve presents the fluorescence intensity of the Wx-Ki-4(scFv) proteins after adding and co-incubation with 2 μg Ki-4 full length antibody and the purple curve after the addition of 5 μg Ki-4 full length antibody.

An additional competitive flow cytometry analysis was performed in order to prove that the specificity of Wx-Ki-4(scFv) binding was not impaired by the presence of the W-tags (Figure 6). Gradually higher concentrations of the bivalent monoclonal Ki-4 full length antibody were incubated on L540cy cells simultaneously with the Wx-Ki-4(scFv) proteins to replace the monovalent single-chain fragment variable. We were able to show that the fluorescence intensity, indicating the specific binding ability of the Ki-4(scFv) fusion protein, decreases with an increasing concentration of Ki-4 full length antibody resulting in a lower fluorescence intensity and a curve shift to the left. Binding of the Ki-4(scFv) and W1-Ki-4(scFv) is stronger, meaning higher concentrations of the Ki-4 full length antibody were necessary to suppress single-chain binding, than the binding of the W2-Ki-4(scFv) and W3-Ki-4(scFv). It can be concluded that the Wx-Ki-4(scFv) fusion proteins still possess active and specific binding ability. Although, the binding specificity of the fusion proteins is not impaired, a higher number of tryptophan residues result in a decrease of the binding activity. This was shown by deletion of the Ki-4(scFv) binding signal with a lower concentration of full length antibody for W2 and W3 (Figure 6).

### Extension of the W-tag concept to a second scFv antibody

In order to demonstrate that the W-tagging concept is generally applicable and not unique to the original model antibody the on-line measurement experiments were repeated using a second, unrelated antibody. We chose M12(scFv), which is derived from a human monoclonal antibody that recognizes the antigen Muc1 [19]. This second antibody was fused to the five different W-tags and was expressed in *E.coli* under the cultivation conditions described above for Ki-4(scFv). The fluorescence behavior of this antibody and the W-tags were similar to that of the original model (Additional file 1).

## Discussion

The simultaneous evaluation of cell growth and target protein quantification without disturbing the actual cultivation process is a huge challenge when screening large numbers of cultures in parallel and producing recombinant proteins on a large-scale. Fermentation in MTPs [3,23] is a convenient method to validate the production efficiency of, for example, enzymes and pharmaceutically relevant target proteins. Thus, the best performing clone can be identified by monitoring cultural growth using surrogate indicators such as optical density (OD_600_), oxygen transfer rate (OTR) or culture fluorescence [3,24]. Product quantification is usually achieved by sampling and off-line analysis e.g. by measuring enzymatic activity [4] or performing an enzyme linked immunosorbent assay (ELISA). Nevertheless, non-invasive on-line detection is preferable. This may be achieved by measuring intrinsic protein fluorescence or by expressing the target protein tagged with a fluorescent marker [4], e.g. a conventional fluorescent protein such as GFP [5] or a FMN-based proteins [6,7]. Major drawbacks of GFP and its derivatives are the large size, which may increase metabolic stress, inhibit protein folding or interfere with protein secretion into the supernatant, the dependence on a fully aerobic environment and the delay between expression and fluorescence detection [5,7-9]. FMN-binding proteins are approximately half the size of GFP and are not oxygen dependent, but they may still cause metabolic stress and interfere with the folding of small target proteins.

In contrast, the short-chained W-tags we developed are comparatively small (5-11% of the size of GFP by molecular weight). Tryptophan has the ability of auto-fluorescence and does not need oxygen to mature. Therefore we presume a functionality of the W-tags even under oxygen-limited conditions. That means they are suitable for the multiplex parallel on-line analysis of cultivated cells producing fusion proteins without any of the disadvantages caused by larger tags. We developed energetically ideal tags *in silico* and then inserted the corresponding sequences inflame with the coding sequence for the Ki-4(scFv). We used a tightly regulated inducible pET expression system [21] so that we could compare non-induced cultures lacking the recombinant protein production to cultures expressing different W-tagged forms of the same recombinant antibody. The induced cultures showed a strong over-expression of the Wx-Ki-4(scFv) fusion proteins where the different tags could be distinguished according to the intensity of the fluorescence signal compared to the untagged protein. The calculated product fluorescence intensity increases with the number of tryptophan residues from EC over W1 to W3. However, W4 and W5 do not follow this trend. Even though they comprise a higher number of tryptophan residues they had slightly weaker fluorescence intensity than W3. Due to the high hydrophobicity from the accumulated tryptophan residues, the fusion proteins might preferentially interact with cell membranes, which may result in a partial quenching of fluorescence intensity. Rather than hydrophobicity the formation of exciplexes presents another probable explanation. Exciplexes are photo induced electron-transfer reactions, which occur during bimolecular encounter of an excited molecule and a quencher [25,26]. The dense packing of tryptophan residues in the W4- and W5-tag as well as the increasing target product concentration towards the end of the fermentation may support exciplex formation. Quenching and, therefore, decreasing fluorescence signal intensity may be a consequence of that effect.

During MTP fermentation, we monitored slightly increased scattered light and tryptophan fluorescence signals, even though no bacterial growth was detected. The MTPs were sealed with a gas permeable membrane resulting in some degree of evaporation. Due to the evaporation the medium components became more concentrated and, therefore, the signals (especially the scattered light signal) increased.

The binding activity of Ki-4 (scFv) [18] was not affected by the presence of the W-tags, as we showed that all the different W-tagged versions of the Ki-4(scFv) were able to bind the L540cy cell line expressing the cognate antigen. Identical results for the on-line analysis in MTPs were obtained with a second, unrelated antibody, demonstrating that the W-tagging concept is generally applicable.

Our evaluation of the W-tags revealed that fusion proteins were not secreted into the medium, making them difficult to retrieve and quantify. This was probably caused by the hydrophobicity of the tryptophan residues and their placement on the outer shell of the protein loop. The W-tagged fusion proteins with four and five tryptophan residues (W4, W5) could not be detached from the bacterial pellet and were, therefore, not analyzed by either flow cytometry or 2D-fluorescence intensity scanning. However, protein extraction with TES buffer containing EDTA resulted in the partial release of proteins tagged with W1, W2 and W3 from the cell pellet. As a result, whereas more tryptophan residues generated a stronger tryptophan fluorescence signal, they also made it more difficult to concentrate the tagged protein in the cell lysate.

Purification of the tagged recombinant proteins by immobilized metal ion chromatography (IMAC) was unsuccessful, probably indicating that the His_6_-tag was obscured by the W-tag (which is larger and immediately adjacent) or that EDTA in the lysis buffer could not be removed from the protein solution by desalting. EDTA can form complexes with Co^2+^ ions and elute them from the IMAC resin, thus preventing the capture of His_6_-tagged proteins. If steric hindrance is preventing protein recovery by IMAC, then potential solutions include switching the order of the tags, separating them with an intervening linker, or appending them to different termini. If the presence of EDTA is preventing sufficient recovery, then a potential solution would be to replace the His_6_-tag-with a FLAG epitope [27]. This would also prevent EDTA disrupting downstream purification strategies involving the use of Ni-NTA or talon columns. The use of stronger lysis buffers with different detergents might also improve the recovery of purified target protein. Bearing in mind that the W-tags described in this article are prototypes, it is also possible that their performance could be improved by additional structural modifications. Although, our W-tags were designed for on-line product detection, the hydrophobicity of tryptophan could also be exploited as a strategy to purify target fusion proteins using an aqueous two-phase system [15,16].

The excitation and emission properties of tryptophan are strongly influenced by other compounds in the solution [12]. We used an excitation of 280 nm for the on-line monitoring and recorded fluorescence emission at 350 nm, but these wavelengths did not map onto the maximum tryptophan fluorescence 2D-analysis in crude extracts (Figure 4). Instead, maxima were observed at 292 nm (excitation) and 338 nm (emission). But we have to consider that the surrounding solution during the cultivation and the off-line measurement of the 2D-analysis is not the same in relation to e.g. ionic strength and media composition. Differences in the optimal excitation and emission wavelengths does not in principle affect the value of the data, and should be determined on a case-by-case basis for individual fusion proteins, media compositions, pH values and other parameters.

Statistically, every cell contains proteins that include tryptophan residues, so the fluorescence signal produced by induced cultures expressing W-tagged recombinant proteins represents a mixture of the product signal and the biomass signal. It is, therefore, necessary to cultivate induced and non-induced cells in parallel to determine the biomass signal and subtract that from the total fluorescence to calculate the signal for the tagged recombinant protein (Figure 3E). This strategy would nominally double the number of assays required and, thus, the number of wells used in MTPs. However, as we have shown, the correlation between fluorescence intensity and scattered light intensity for the non-induced cultures was the same for all fusion protein variants. Therefore, it should be sufficient to cultivate just one of the variants as a non-induced control to determine biomass fluorescence. The fluorescence intensity of each fusion protein is also substantially greater than that of the corresponding untagged target protein (Figure 3F), even when that protein contains multiple tryptophan residues [12,28,29]. Due to the advantages of the W-tags, their high fluorescence intensity, future research should focus on optimizing the presented W-tags for being secreted or for purification application. Ways to release the W-tags from the bacterial pellet still has to be improved.

## Conclusions

In conclusion, our novel W-tags are ideal for the non-invasive monitoring of recombinant protein accumulation, lacking the main drawbacks of conventional fluorescent proteins such as GFP or FMN-based proteins such as evoglow®. The low-molecular-weight W-tags can be used to monitor online protein production. The binding specificity of our recombinant fusion protein is not affected. However, the binding activity of the recombinant protein fused to one of the W-tags decreases slightly with increasing number of tryptophan residues in the W-tag. W-tags, therefore, represent a valuable generally applicable alternative to GFP and its derivatives for the rapid on-line qualitative measurement of target recombinant proteins.

## Methods

### Bacterial strains and gene sequences

General cloning was carried out using *E.coli* strain XL1-Blue (Stratagene, La Jolla, CA). The W-tagged fusion proteins were expressed in *E.coli* strain BL21 Rosetta 2 (DE3) (Novagen, Nottingham, UK). The W-tags were synthesized by GENEART (Regensburg, Germany). These DNA sequences were delivered in the plasmid pMA_(amp)_ and pCR4Blunt-TOPO_(amp,kan)_ containing all necessary restriction sites, His_6_-tag, GS-linker and cleavable enterokinase site.

### Cell culture

The Hodgkin lymphoma derived, CD25^+^ and CD30^+^ cell line L540cy [30] was provided by Fraunhofer IME, Aachen, Germany, and was cultivated in complex RPMI 1640 Glutamax medium supplemented with 10% fetal calf serum, 50 μg/mL penicillin and 100 μg/mL streptomycin (all supplied by GIBCO BRL). All cells were cultured at 37°C in a 5% CO_2_ atmosphere.

### Cloning the expression constructs

The expression cassettes were released from the GENEART cloning vectors by digestion with NcoI and HindIII, then ligated into the expression vector pET-27b+ (Novagen, Nottingham, UK) which had been cut at the same sites. The complete expression cassette comprised a *pel*B leader, W-tag (W1–W5), His_6_-tag, GS-linkers and a cleavable enterokinase site DDDDK (Figure 2A). The vector also contained a Kanamycin resistance gene, a pBR322 origin of replication and the lactose repressor gene (*lacI*) regulated by a T7 promoter. The Ki-4(scFv) gene [18] was introduced as an in frame fusion downstream of the expression cassette using SfiI and NotI. Plasmid DNA was isolated using Nucleo Spin plasmid kits from Macherey & Nagel (Düren, Germany). After restriction digest (all enzymes purchased from New England Biolabs, NEB, USA), the resulting fragments were separated by horizontal agarose gel electrophoresis and purified with the QIAquick Gel Extraction Kit from Qiagen (Hilden, Germany). Cloning of the plasmid vectors was performed by standard methods [31] with T4 DNA ligase.

Expression was induced via the *lac* operator [17] with isopropyl-β-D-1-thiogalactopyranoside (IPTG). The empty pET-27b+ vector was used as the negative control (NC) and the pET-Ki-4(scFv) vector was used as the expression control (EC) and to monitor background fluorescence.

### Expression and release of Wx-Ki-4(scFv) fusion proteins

Large quantities of the target fusion proteins and the expression control proteins were produced by bacterial fermentation in MTPs and 1-L Erlenmeyer flasks by maintaining the maximum oxygen transfer rate in both systems (calculation not shown) [32,33]. The Erlenmeyer flasks contained 80 mL modified Wilms-Reuss synthetic medium [34] containing 20 g/L glucose and 50 μg/mL Kanamycin. After 4 h, the bacterial cultures were induced with 1 mM IPTG and cultivated at 37°C for another 6 h. After a total of 10 h, the cells were centrifuged at 4000 x g, 4°C, 30 min. The pellets were resuspended in 8 mL 1 x TES buffer (40% sucrose, 50 mM Tris, 1 mM EDTA, pH 8.0) containing one ‘Complete’ protease inhibitor tablet (Roche Diagnostics, Mannheim, Germany) for each 50 mL, and then incubated for 15 min. We then added 12 mL 0.2 x TES buffer and sonicated the suspension five times on ice for 60 s at 70% amplitude (Sonoplus, Bandelin, Berlin, Germany). The periplasmic fraction was recovered after centrifugation at 15,000 x g, 4°C, 30 min. The crude lysate was passed through a desalting column (GE Healthcare, Munich, Germany) to exchange the buffer with PBS and remove EDTA. The protein content of the solution was determined by Western blot using AIDA analysis software.

### SDS-PAGE and Western blot analysis

SDS-PAGE was carried out in duplicate with BioRad electrophoresis system at 100-150 V using a 12% polyacrylamide gel. Whole cell extracts were completely denatured by heating and we loaded 100 μg protein per lane. Separated protein bands were stained with Coomassie Brilliant Blue (SERVA, Heidelberg, Germany) and the proteins in the duplicate gel were transferred to a nitrocellulose membrane (GE Healthcare, UK), which was blocked with 2% milk powder (Campina, Netherlands) in 1 x PBS. The fusion proteins were detected by using a primary α-poly-His mouse IgG (H1029, Sigma) which was then detected with a goat α-mouse IgG peroxidase secondary antibody (A2554, Sigma). The signal was developed with 3,3^′^-diaminobenzidine tetra hydrochloride (DAB, Sigma) activated with 30% H_2_O_2_.

### Fermentation in microtiter plates

We used 96-well MTPs (lumox, Greiner bio-one, Kremsmünster, Austria) for cultivation and sealed the plates with a gas permeable membrane (AB-0718; Agene, Epsom, UK) to allow aeration while minimizing evaporation. Bacterial pre-cultures from all clones (NC, EC, Wx-Ki-4(scFv)) were inoculated from cryo cultures grown over night in 10 mL modified Wilms-Reuss synthetic medium supplemented with 20 g/L glucose in 250 mL shake flasks sealed with cotton plugs at 37°C, a shaking frequency of 350 rpm and 50 mm shaking diameter. For the main cultures the clones were transferred to MTPs (200 μL per well) and incubated at 37°C shaking at 950 rpm and a shaking diameter of 3 mm. The OD_600_ for all variants was adjusted to 0.1. The cultures were induced with 1 mM ITPG at the beginning of the exponential phase (t = 3.2 h). The experiments were conducted in a temperature-controlled room under an aerated hood with humidified air to minimize evaporation.

### On-line measurement

Cultivation parameters were measured using an adapted BioLector® fiber optic monitoring device [2,3]. The adapted device included a modified orbital shaker (Kühner, Basel, Switzerland), an x-y linear motion unit (BMG, Offenburg, Germany), a fluorescence spectrophotometer with filter wheels (Fluostar, BMG, Lab Technologies, Offenburg, Germany) and a computer. All the filters had an optical band width of 10 nm. The biomass was quantified using 180° backscattered light at 620 nm. Tryptophan was excited at 280 nm and detected at an emission wavelength of 350 nm. Both measurements were obtained while the cultures were shaken continuously.

### Flow cytometry

L540cy cell suspensions (2 × 10^5^ cells/mL) were washed with ice-cold PBS, incubated on ice for 1 h with crude extracts containing 2 μg per sample of the recombinant proteins, washed twice with PBS and incubated on ice for 30 min with an anti-His_5_ antibody conjugated to AlexaFluor488 (QIAGEN). The fluorescence signal from the bound proteins was measured by flow cytometry using a FACScalibur (BD Biosciences, NJ) and the data were analyzed using WinMDI (version 2.9). The geometric mean values of flow cytometry measurements were displayed as percent changes in relation to Ki-4(scFv) expression control (100%) and were analyzed by Cell Quest Pro software (BD Biosciences, NJ).

### Competitive flow cytometric analysis

Binding specificity after W-tag fusion to the Ki-4(scFv) was determined by competitive flow cytometry using hybridoma derived monoclonal full length antibody Ki-4 (mAB Ki-4) (from Fraunhofer IME, Aachen) as competitive entity towards Ki-4(scFv). The bivalent structure of the mAB Ki-4 was meant to replace the monovalently bound Ki-4(scFv) proteins on the L540cy cells. Therefore, L540cy cell suspensions (2 x 10^5^ cells/mL) were washed with ice-cold PBS, incubated on ice for 1 h with Ki-4(scFv), W1-Ki-4(scFv), W2-Ki-4(scFv) and W3-Ki-4(scFv) crude extracts in combination with the mAB Ki-4 using gradually higher total concentrations of 0 μg, 0.5 μg, 2 μg and 5 μg mAB Ki-4 per sample. Tubes were washed twice with PBS and incubated on ice for 30 min with an anti-His_5_ antibody conjugated to AlexaFluor 488 (QIAGEN). The fluorescence signal was measured and analyzed as indicated above.

### Two-dimensional fluorescence intensity scans

We analyzed the fluorescence intensity of 200 μL crude extract in 96-well MTPs (lumox, Greiner Bio-One, Germany) by two-dimensional fluorescence scanning. The W-tag fusion proteins containing one to three tryptophan residues and EC were diluted to 3 μg/mL in 1 x PBS and the fluorescence intensity was measured at excitation wavelengths of 250–300 nm and emission wavelengths of 300–400 nm using a FluoroMax-4P (Horiba Jobin Yvon, USA) with a Y-shaped optical fiber.

## Abbreviations

a.u: Arbitrary units; DOT: Dissolved oxygen tension; EC: Expression control; EDTA: Ethylene-diamine-tetraacetic acid; FbFP: FMN-based fluorescent protein; FMN: Flavin mononucleotides; GFP: Green Fluorescent Protein; IPTG: Isopropyl β-D-1-thiogalactopyranoside; mAB: Monoclonal full length antibody; MFI: Mean fluorescence intensity; MTP: Microtiter plate; NC: Negative control; OD: Optical density; scFv: Single-chain fragment variable; W: Tryptophan (one letter code for amino acid); YFP: Yellow Fluorescent Protein.

## Competing interests

The authors declare no conflict of interest.

## Authors’ contributions

Esther Gartz and Eva-Maria Siepert designed this study. Esther Gartz carried out the cultivation experiments, the data analysis as well as the 2D-fluorescence scans. Eva-Maria Siepert carried out the cloning and the off-line molecular biological analysis as well as the functionality studies. Esther Gartz and Eva-Maria Siepert drafted the manuscript. Heinrich Delbrück designed and calculated the different W-tag versions and drafted the appropriate paragraph. Jochen Büchs, Mehmet K. Tur and Stefan Barth supervised the study and assisted in drafting the manuscript. All authors read and approved the final manuscript.

## Supplementary Material

Additional file 1 **On-line detection of biomass formation and production of W-tag labeled M12(scFv).** On-line fermentation signals measured with a modified BioLector^®^ device during cultivation of *E.coli *BL21 Rosetta 2 (DE3) expressing the M12 single-chain variable fragment in modified Wilms-Reuss medium with 20 g/L glucose using a 96-well microtiter plate. The intensities of (A,B) the scattered light (ex:620 nm/ em:-) and (C,D) tryptophan fluorescence (ex:280 nm/em:350 nm) were measured for the non-induced and induced cultures (induction with 1 mM IPTG: vertical dash-dotted line after 3.2 h of cultivation). (E) For the non-induced cultures (no product), tryptophan fluorescence is plotted versus scattered light intensity (symbols). The appropriate fit is given as the continuous line (power function: fluorescence intensitycalc = - 4190·10^8^· [scattered light intensity_meas_]^1,35^ + 6415); the plot of the residues between the calculated and the measured fluorescence is displayed within the diagram as inserted. (F) Product fluorescence intensity resulting from the total fluorescence of the induced cultures derived from product and biomass minus the fluorescence originated from biomass calculated from the fit (E) for the uninduced cultures. Click here for file
