# Supplementary figures and images for: Short-chain fluorescent tryptophan tags for on-line detection of functional recombinant proteins
Source: BMC Biotechnol. 2012 Sep 21;12:65. doi: 10.1186/1472-6750-12-65 (PMC3544578; doi:10.1186/1472-6750-12-65)

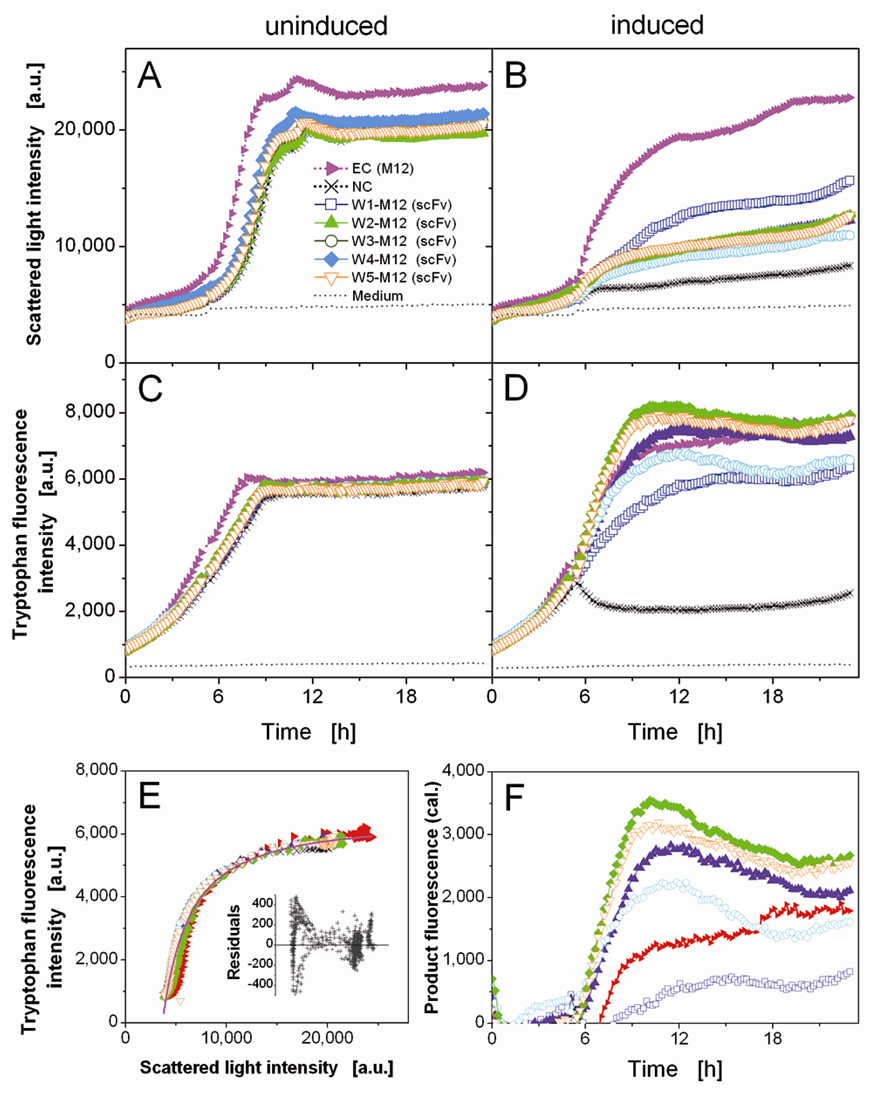

Supplement: Additional file 1 — On-line detection of biomass formation and production of W-tag labeled M12(scFv). On-line fermentation signals measured with a modified BioLector® device during cultivation of E.coli BL21 Rosetta 2 (DE3) expressing the M12 single-chain variable fragment in modified Wilms-Reuss medium with 20 g/L glucose using a 96-well microtiter plate. The intensities of (A,B) the scattered light (ex:620 nm/ em:-) and (C,D) tryptophan fluorescence (ex:280 nm/em:350 nm) were measured for the non-induced and induced cultures (induction with 1 mM IPTG: vertical dash-dotted line after 3.2 h of cultivation). (E) For the non-induced cultures (no product), tryptophan fluorescence is plotted versus scattered light intensity (symbols). The appropriate fit is given as the continuous line (power function: fluorescence intensitycalc = - 4190·108· [scattered light intensitymeas]1,35 + 6415); the plot of the residues between the calculated and the measured fluorescence is displayed within the diagram as inserted. (F) Product fluorescence intensity resulting from the total fluorescence of the induced cultures derived from product and biomass minus the fluorescence originated from biomass calculated from the fit (E) for the uninduced cultures. [file 1472-6750-12-65-S1.tiff]
